# Alternative Splicing of Exon 23a in Neurofibromatosis Type 1 Pre‐mRNA: Its Contribution to the Protein Structure and Function of Neurofibromin

**DOI:** 10.1002/wrna.70021

**Published:** 2025-08-14

**Authors:** Annabelle G. Elsner Pacheco, Hua Lou

**Affiliations:** ^1^ Department of Genetics and Genome Sciences Case Western Reserve University School of Medicine Cleveland USA; ^2^ Department of Genetics and Genome Sciences, Center for RNA Science and Therapeutics, Case Comprehensive Cancer Center Case Western Reserve University School of Medicine Cleveland USA

**Keywords:** alternative splicing, exon 23a, NF1

## Abstract

The neurofibromatosis type 1 (*NF1*) gene has 61 exons. The major alternative exon in *NF1* pre‐mRNA is exon 23a. Skipping and inclusion of this exon produce isoform I and isoform II neurofibromin, respectively. When the alternative exon was discovered in 1993, several experiments conducted in yeast and human cell lines quickly led to the conclusion that inclusion of this exon reduced the RasGAP function of the neurofibromin protein by 5–10‐fold. Since then, research efforts on this seemingly important alternative splicing event have been sporadic, leaving many important questions unanswered, until after 2020 when several important papers related to the structure and function of exon 23a have been published. Two major advancements have been made. First, the cryo‐EM structures of the full‐length neurofibromin, of both isoforms, have been solved. More excitingly, the structure of isoform II neurofibromin that includes exon 23a provides important insight into why this isoform has reduced RasGAP activity. Second, the role of the altered splicing pattern of exon 23a in the development of high‐grade glioma (HGG) has been investigated. In this review, we start with the introduction of alternative splicing of exon 23a, its discovery, differential expression patterns, and regulatory mechanisms that control this alternative splicing event. Next, we discuss the structural differences between the two isoforms which give insight into the differing RasGAP activities. We then review the in vivo biological function of the regulated inclusion of exon 23a, focusing on cognitive behaviors and brain tumor development. Finally, we briefly discuss the future directions of studies on NF1 exon 23a.

This article is categorized under:

RNA Processing > Splicing Regulation/Alternative Splicing

## Graphical abstract

Isoform I and II of neurofibromin differ in the inclusion of exon 23a and their ability to regulate membrane‐bound RAS and consequent downstream pathways. It is predicted that the insertion of exon 23a disrupts trafficking of the protein to the membrane and/or with RAS itself.

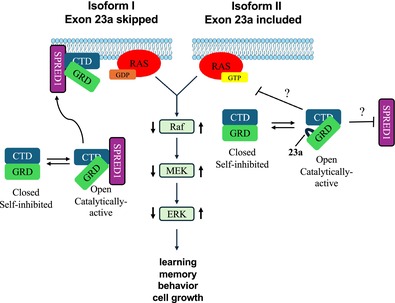

## 1 Introduction

The central dogma of biology states that DNA is transcribed into RNA that is translated into protein, and until the 1940s, biologists believed that each gene encodes one protein (Beadle and Tatum 1941). However, this paradigm was shifted with the discovery of multiprotein complexes, non‐coding RNAs, and alternative splicing. Alternative splicing occurs when exons and introns are differently included, giving rise to different mature mRNA isoforms. When high throughput human genome sequencing revealed in 2003 that humans had only 22,000 genes, the scientific community was shocked because the protein complexity was significantly higher. Alternative splicing offers an explanation for how one gene can encode many different proteins, and it is estimated that up to 94% of human genes undergo alternative splicing (Pan et al. 2008).

Alternative splicing is an important and major pathway for protein diversity, and it also plays fundamental roles in development and highlights biological importance in both healthy and disease physiologies. Protein isoforms resulting from alternative splicing can have differing properties leading to different binding interactions, localization, and activities (Stamm et al. 2005). Differing properties of mRNA isoforms play critical roles in development and cell differentiation. A well‐known example of the role of alternative splicing in development is in the heart. The transition that the heart must make due to the different oxygen and metabolic environments in utero vs. postnatally is made possible, in part, through alternative splicing. During heart development, expression of several RNA binding proteins undergoes drastic changes that regulate splicing. Splicing regulators have been shown to be responsible for maturation of cardiac ventricles, cardiomyocytes, and cardiac fibroblasts following birth by changing splicing patterns of a number of alternative exons to express adult isoforms (Giudice et al. 2014). Cardiac diseases often occur with reversions from the adult to fetal isoforms via downregulation of the RNA binding proteins that regulate splicing (Baralle and Giudice 2017).

Dysregulation of alternative splicing has been reported to play roles in numerous human diseases including cystic fibrosis, spinal muscular atrophy, and neurofibromatosis type I (NF1) (Tazi et al. 2009). Cassette exons, also known as exon skipping, are the most common alternative splicing events in humans, and occur when an exon is differentially included. One example of a cassette exon is exon 23a in the *NF1* gene that plays important biological roles in the function of neurofibromin and is the focus of this review.

## 2 Neurofibromatosis Type I and Neurofibromin

NF1 is a common genetic disease that is estimated to affect 1 in 3100 people worldwide that occurs when one copy of the *NF1* gene has a loss‐of‐function mutation, leaving patients with only one functioning allele of *NF1* (Lee et al. 2023). NF1 patients have an increased risk of developing tumors such as peripheral nerve tumors and plexiform neurofibromas when the second allele of *NF1* is mutated that often progresses into malignancies (Xu et al. 2025). In addition to tumor development, NF1 has a wide variety of clinical manifestations including café au lait spots, Lisch nodules, cardiovascular abnormalities, skeletal defects, and cognitive and behavioral difficulties (Wilson et al. 2021). Some of these symptoms occur due to haploinsufficiency of the *NF1* gene. Manifestations, mechanistic insights, and therapeutic developments for homozygous loss of the *NF1* gene are reviewed elsewhere (Upadhyaya 2011; Li et al. 2020; O'Donohue et al. 2025). This review will focus on the structural, functional, and biological consequences of alternative splicing of *NF1* pre‐mRNA. This alternative splicing event is relevant when NF1 symptoms are caused by *NF1* haploinsufficiency such as learning and behavioral defects but not in tumor development when both alleles of *NF1* are null.

The protein product of *NF1*, neurofibromin, is best characterized as a negative regulator of membrane‐bound RAS, which is critically involved in cellular proliferation, differentiation, and apoptosis (Scheffzek and Shivalingaiah 2019; Zinatizadeh et al. 2019; Chaker‐Margot et al. 2022). Neurofibromin is a RAS GTPase‐activating protein (RasGAP) that acts as a negative regulator of RAS by accelerating its low intrinsic rate of GTP hydrolysis of GTP‐bound RAS to its inactive form, GDP‐bound RAS (Martin et al. 1990). Loss‐of‐function mutations to the GTPase‐activating‐protein‐related domain (GRD) can cause dysregulation of RAS and lead to unregulated cell proliferation through many of the RAS pathways (Xu et al. 2025).

## 3 *NF1* Alternative Splicing

**FIGURE 1:**
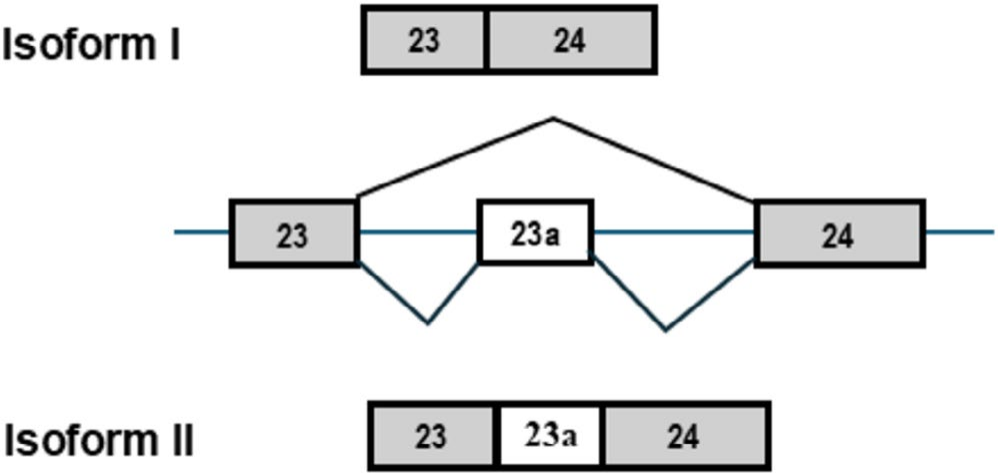
Alternative splicing of *NF1* exon 23a leads to the production of two major isoforms of neurofibromin. Schematic showing that isoform I of *NF1* results from the skipping of exon 23a, while the inclusion of the alternatively spliced exon results in isoform II.

There are two major isoforms of *NF1* pre‐mRNA resulting from alternative splicing of exon 23a, which differ in their RAS‐GTP activity. It should be noted that in the current *NF1* nomenclature, exon 23a is renamed as exon 31. In this review, we will use its original name, exon 23a. Isoform I, the first characterized isoform, does not include exon 23a (Figure 1). Isoform II includes exon 23a that is 63 nucleotides (nt) in length and encodes 21 basic amino acids. Exon 23a is located in the GRD of neurofibromin that is responsible for the negative regulation of RAS pathways. Both isoforms remain functional as RasGAPs; however, the inclusion of exon 23a in isoform II reduces the RasGAP function by up to 10‐fold (Andersen et al. 1993; Hinman et al. 2014; Nguyen et al. 2017).

Alternative splicing of NF1 exon 23a occurs in a tissue‐ and developmental stage‐specific manner, indicating that each isoform has distinct and important functions. Isoform I, with exon 23a skipped, is expressed predominantly in neurons of the nervous system, and isoform II, with exon 23a included, predominates in most other tissue types, including glial cells, lung tissue, and the adrenal gland (Mantani et al. 1994; Gutmann et al. 1999). Interestingly, exon 23a shows a higher inclusion incidence in primary brain tumors (Suzuki et al. 1991). Additionally, studies in mouse and chicken show that during embryonic development, there is a switch made from primarily exon 23a inclusion to skipping at E10 and E7, respectively (Baizer et al. 1993; Huynh et al. 1994). The clear and conserved splicing patterns in tissues and during development point to the conclusion that each isoform has differing functions (Huynh et al. 1994).

To add to the evidence that each isoform has separate functional significance, several studies have demonstrated that having both isoforms in a balance is biologically important. In cultured cells, changing the relative abundance of the two isoforms in PC12 cell culture with antisense oligonucleotides results in dysregulated neuronal differentiation through the neurofibromin‐ and RAS‐mediated MAPK/ERK and cAMP/PKA signaling pathways (Biayna et al. 2021). The biological role of NF1 exon 23a in vivo is discussed in section 7.

## 4 Cryogenic Electron Microscopy (Cryo‐EM) Structures of Both Neurofibromin Isoforms Reveal Structure–Functional Differences in RasGAP Abilities

**FIGURE 2:**
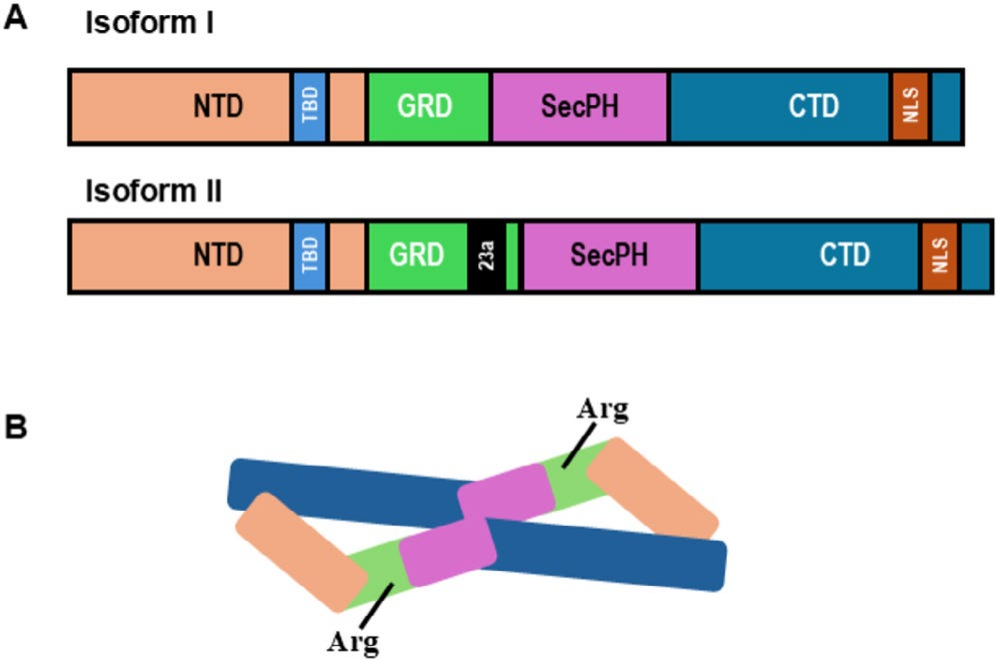
Neurofibromin, the protein product of *NF1*, is a large multidomain protein that forms a lemniscate‐shaped homodimer. (A) A diagram highlighting that isoforms I and II share common NTD (N‐terminal domain), TBD (tubulin binding domain), SecPH, NLS (Nuclear localization signal), and CTD (C‐terminal domain). GRD (GTPase‐activating‐protein‐related domain) is similar except that isoform II results from the alternative splicing of exon 23a and the insertion of 21 basic amino acids. (B) Schematic representation of the neurofibromin homodimer highlighting the catalytically active arginine residues and GRD near the central interface of the dimer.

Neurofibromin is a large multidomain protein with a mass of 319 kDa. Isoforms I and II contain 2818 and 2839 amino acids, respectively (Figure 2a). The protein exists in a lemniscate‐shaped high‐affinity dimer (Lupton et al. 2021) (Figure 2b). Near the central interface of the homodimer is the GRD (Sherekar et al. 2020). In the GRD, an arginine finger is essential for stabilizing and orienting Q61 in RAS for nucleophilic attack of the gamma phosphate of GTP (Bergoug et al. 2020). Its RasGAP activity occurs in membrane‐bound RAS. Sprouty‐related EVH1 domain containing 1 (SPRED1) is a well‐known interacting partner with neurofibromin that recruits the homodimer from the cytosol to the plasma membrane where it can interact with membrane‐bound RAS (Stowe et al. 2012; Chaker‐Margot et al. 2022).

Cryo‐EM structures have revealed that neurofibromin isoforms exist in two functional states: an open active state and a closed self‐inhibited state. In the closed state, the GRD domain is buried in the cleft formed at the central interface, where its binding to RAS is sterically inhibited (Lupton et al. 2021). In the open conformation, there is a rotation of one of the protomers, turning the GRD away from the central interface of the dimer, allowing access to RAS. Neurofibromin in the open conformation has two significant structural features that provide insight into its higher RAS GTPase abilities. The first is that the interacting site of neurofibromin and RAS is turned outward, where there is steric space for RAS. The second is that the arginine finger is in the active site and close enough to RAS's Q61 for the stabilization that is required for catalysis (Naschberger et al. 2021). However, in the closed conformation, the GRD‐RAS access site is turned inward and blocked by the central interface of the homodimer. Additionally, the essential arginine is not able to access the catalytic glutamine, leaving the homodimer unable to catalyze the phosphorylation of GTP.

Both open and closed conformations of neurofibromin have been solved via cryo‐EM for each isoform, and both isoforms exist in an equilibrium between the two states. To date, nucleotide binding has been shown to stabilize the open conformation of neurofibromin isoform I, and zinc ion binding stabilizes the closed conformation of isoform II (Naschberger et al. 2021; Chaker‐Margot et al. 2022). It remains unclear whether these mechanisms are shared between the isoforms. However, it is clear that the functional state of each is highly regulated, but the intricacies of these mechanisms still need to be elucidated (Naschberger et al. 2021; Chaker‐Margot et al. 2022).

**FIGURE 3:**
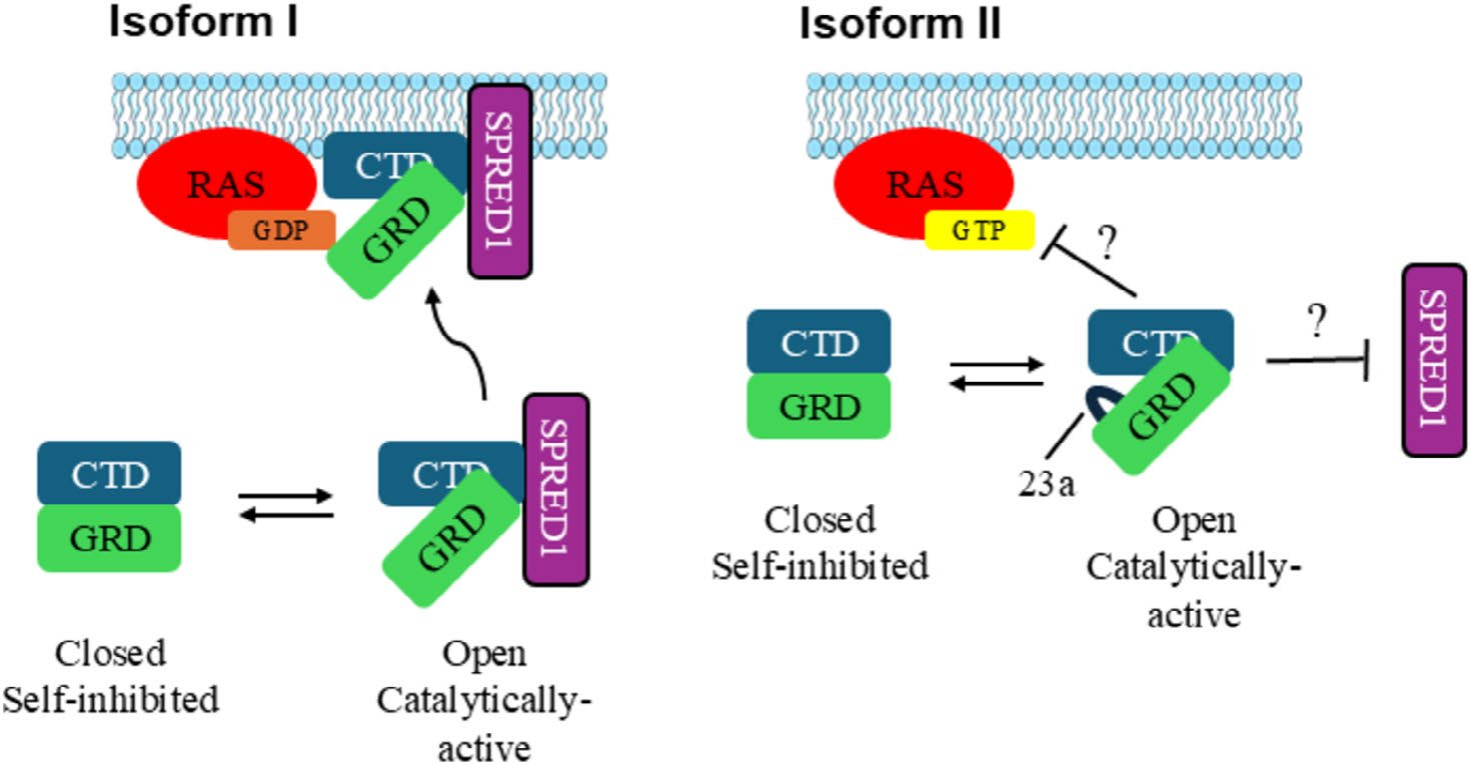
Neurofibromin isoform I and II exist in an equilibrium between the open and closed states but have differing RAS‐GAP abilities. Schematic representation of the closed and open states of the neurofibromin homodimer. For both isoforms, in the closed and self‐inhibited state, the GRD (GAP related domain) is buried in the central interface with the CTD (C‐terminal domain) where it is unable to bind RAS. However, in an open active state, the GRD rotates, leaving it accessible to RAS. (A) Isoform I neurofibromin is able to interact with SPRED1 (Sprouty related EVH1 domain containing 1) to be recruited to the membrane where it can hydrolyze GTP‐bound RAS. (B) For isoform II neurofibromin, the interaction of neurofibromin with SPRED1 and/or membrane‐bound RAS‐GTP is perturbed.

In the cryo‐EM structure of isoform II, the 21 amino acid insertion of exon 23a forms a lysine‐rich loop, which in the open conformation is proximal to where neurofibromin binds RAS and SPRED1. While it is not clear if the interaction is direct, it is suggested that the insertion of this positively charged and partially disordered loop could interfere with these interactions (Yan et al. 2020; Naschberger et al. 2021). This would imply that either neurofibromin type II has lost access to membrane‐bound RAS directly or with its membrane recruiter, SPRED1. Either inhibited recruiting to the membrane or perturbed interaction with RAS would lead to reduced catalytic activity of the GRD and increased RAS activity (Yan et al. 2020; Naschberger et al. 2021). These hypotheses have yet to be tested; however, this finding leads to a tantalizing potential structural explanation for the drastically reduced RasGAP activity of neurofibromin type II (Figure 3).

## 5 Regulation of Exon 23a Alternative Splicing

**FIGURE 4:**
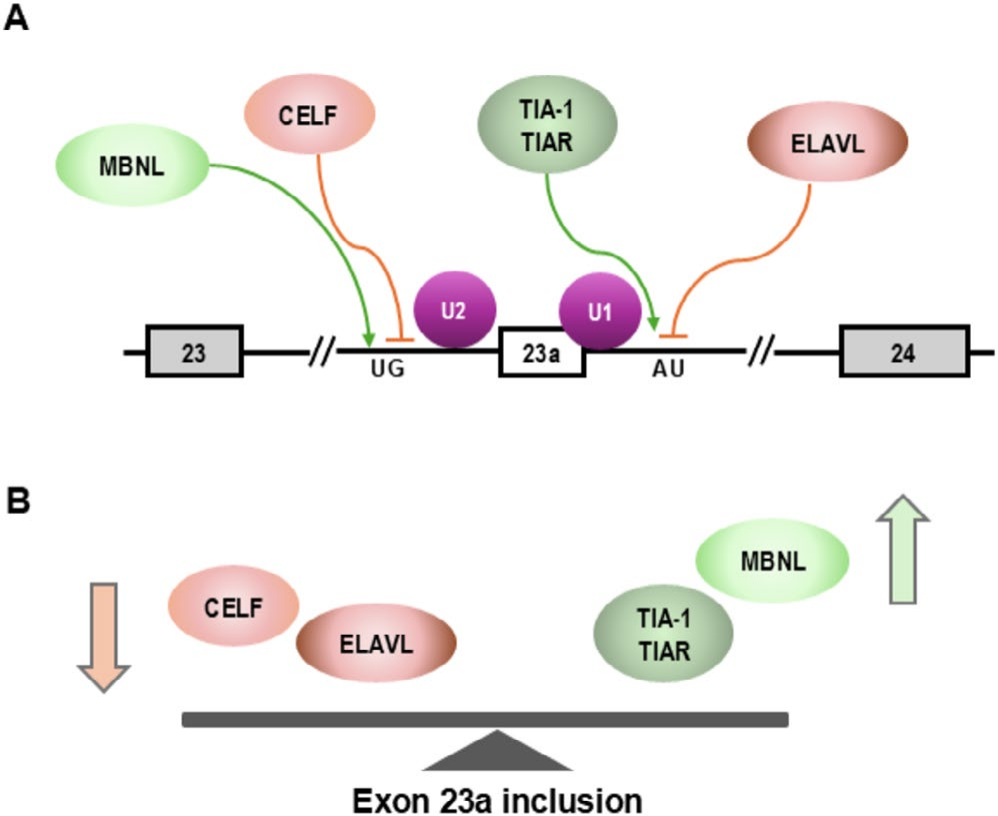
Splicing regulators of NF1 exon 23a alternative splicing. (A) Schematic showing CELF (CUG‐binding protein) as negative regulators (pink circles) of exon 23a inclusion that act by binding to UG rich elements upstream of exon 23a and prevent binding of other splicing factors. MBNL (Muscleblind‐like family proteins) binds to the same region and acts as positive regulators (green circles) of exon 23a inclusion. ELAVL (Embryonic Lethal Abnormal Vision‐Like family) proteins are negative regulators (pink circle) of 23a inclusion and thus cause skipping by binding to intronic splicing silencer elements that are AU rich. TIA‐1 and TIAR proteins lead to inclusion of exon 23a when they bind the same region and block binding of ELAVL proteins. (B) Modulating the expression of these splicing factors have been shown to cause shifting of isoform type expression and regulate alternative splicing of exon 23a.

Alternative splicing of *NF1* exon 23a is strongly regulated, and four families of proteins have been discovered to modulate its inclusion level; the CELF and ELAVL proteins are negative regulators of exon 23a inclusion while MBNL and TIA‐1/TIAR proteins promote inclusion of the exon (Zhu et al. 2008; Fleming et al. 2012) (Figure 4). Our laboratory has shown that knockdown of CUG‐binding protein and embryonic lethal abnormal vision like factor proteins (CELF), which traditionally serve as important alternative splicing regulators, promotes skipping of exon 23a. In vitro UV crosslinking and immunoprecipitation experiments show that CELF proteins and U2AF65, which is an essential splicing factor that interacts with the polypyrimidine‐tract sequence at the 3′ splice site, bind in the same area. These experiments suggest that when CELF proteins bind upstream of exon 23a, the binding of U2AF65 is blocked, and splicing is prevented (Barron et al. 2010).

Embryonic Lethal Abnormal Vision‐Like family proteins (ELAVL), called Hu proteins in our previous publications, negatively regulate exon 23 inclusion via two mechanisms. The first is by binding the intronic AU‐rich sequences flanking exon 23a and blocking the binding of U1 and U6 snRNPs to the 5′ splice site (5′ ss) to coordinate splicing (Zhu et al. 2008). Secondly, we have shown that ELAVL proteins interact with histone deacetylase 2 to negatively regulate its deacetylation abilities, which can modulate gene expression and transcriptional speed. Reduced histone acetylation downstream of the *NF1* gene increases the speed of RNA polymerase II, which promotes the skipping of exon 23a (Zhou et al. 2011).

One family of positive regulators of exon 23a inclusion is the Muscleblind‐like (MBNL) protein family. We first identified the MBNL motif UGCUGU in the intron upstream of exon 23a and discovered that MBNL binds to overlapping motifs in this region and results in exon 23a inclusion. Interestingly, we found that the ratio of MBNL and CELF was important in determining the inclusion status of exon 23a. Overexpression experiments and binding analysis suggest that there is antagonistic regulation of exon 23a from MBNL and CELF proteins (Fleming et al. 2012).

The second family of known positive regulators of exon 23a inclusion are TIA‐1 and TIAR proteins. Like ELAVL proteins, TIA‐1 and TIAR proteins bind to U rich sequences in the 5′ ss. TIA‐1 and TIAR compete with Hu proteins for binding sites. When Hu proteins are bound, they suppress exon 23a inclusion by decreasing the binding of U1 and U6. However, if these sites are bound by TIA‐1 and TIAR, binding U1 and U6 are enhanced to clearly define 5′ ss (Zhu et al. 2008).

## 6 Deep Intron Mutations in the *NF1* Gene Both Upstream and Downstream of Exon 23a

More than 3300 pathogenic genetic variants have been identified in the *NF1* gene. Of these variants, up to 30% affect splicing of the *NF1* transcript (Koczkowska et al. 2023). Although no variants have been found at the splice site sequences on both sides of exon 23a, a recent study examined more than 8000 NF1 patients at the Medical Genomics Laboratory at the University of Alabama at Birmingham and identified 9 deep intron variants on either side of exon 23a (Koczkowska et al. 2023).

In this study, RT‐PCR analysis was carried out using RNA isolated from patient blood samples, and the RT‐PCR products were sequenced to determine how splicing was affected. In the blood, both splicing isoforms are generated with slightly more exon 23a skipped isoform. All nine variants created a splice site‐like sequence, and when paired with another splice site nearby, generated an artificial exon (aka cryptic exon) from the intronic sequences. In most cases, the cryptic exon is spliced to exon 23a to generate a bigger spliced product that includes exon 23a and the cryptic exon. Importantly, for most variants, generation of the cryptically spliced product is at the expense of both exon 23a included and skipped products. Thus, in these cases, both natural *NF1* isoforms were reduced significantly (in several cases, reducing more than half of the level of the wild‐type transcript), resulting in decreased neurofibromin function (Koczkowska et al. 2023). Mechanistically, why cryptic exons are frequently spliced to exon 23a, as opposed to the upstream or downstream exon, is unclear and will be interesting to study.

## 7 Biological Role of the Regulated Expression of Exon 23a In Vivo

The cell type‐ and developmental stage‐specific expression patterns of NF1 exon 23a, combined with the fact that inclusion of this exon in neurofibromin significantly decreases the function of GRD, suggest that this alternative exon plays an important biological role. Given the critical role RAS activity plays in cell proliferation and function in differentiated cells, it is not surprising that disruption of alternative splicing of *NF1* exon 23a contributes to carcinogenesis in brain tumors and defective cognitive functions in mice. In the following sections, we will review the functional role of NF1 exon 23a in regulating cognitive behaviors in mouse models and the oncogenic pathway in glioma in human patients.

### 7.1 Regulating Cognitive Behaviors

In addition to increased risks of developing tumors, the majority of NF1 patients also suffer from cognitive and behavioral problems including deficits in executive function, attention, misperceptions, and language skills (Gutmann et al. 2025). Because RAS activity plays a critical role in brain functions, it is conceivable that the optimal neurofibromin RasGAP function is required to support the normal cognitive and social behaviors in a healthy individual.

To understand how alternative splicing of *Nf1* exon 23a affects biological functions, our laboratory generated a mutant mouse model in which exon 23a was engineered to be always included in all cell types, that is, exon 23a functioning as a constitutive instead of alternative exon (Nguyen et al. 2017). Because this exon is predominantly skipped in the brain in wild type mice, we predicted that the most dramatic phenotype in the mutant mice would be brain‐related. Examination of the active RAS activity in the mutant mouse whole brain did show hugely increased RAS activity (quantification was not performed because the active RAS was not detectable in the wild‐type mouse brain in our assay) (Nguyen et al. 2017).

A battery of behavioral tests was conducted to examine learning, memory, and motor functions of the mutant mice. The mutant mice displayed deficits in learning and memory behaviors. In the T‐maze test, a novelty‐based short‐term spatial learning and memory test, the mutant mice failed to remember the arm that they did not explore when they were first placed in the T‐maze. In the Morris water maze test, a long‐term spatial learning and memory test, the mutant mice showed deficits in acquisition and probe trials. Compared to the wild‐type mice, it took more than twice as long for the mutant mice to reach the hidden platform, while in the probe test after the hidden platform was removed, the mutant mice spent approximately half the time searching for the missing platform, indicating a defective long‐term memory. In the novel object recognition test, the mutant mice had normal short‐term but deficits in long‐term memory. In both cued and contextual fear conditioning tests, the mutant mice showed deficits in extinguishing the freezing response, similar to having post‐traumatic stress disorder (PTSD) (Nguyen et al. 2017). The mutant mice did not exhibit any motor function defects (rotarod, beam walking and sticky paper tests) or exploratory and anxiety behavior (open field test) (Nguyen et al. 2017).

Historically, tumor development has been the most studied aspect of the NF1 clinical problems. The cognitive and behavioral manifestations have been shockingly understudied. To address this gap, the Cognition and Behavior in Neurofibromatosis type 1 (CABIN) task force was established recently (Gutmann et al. 2025). In this regard, the mutant mice we generated will serve as a valuable mouse model to study additional behavioral issues observed in NF1 patients, for example, depressive behaviors, sleep abnormalities, and social interaction difficulties. Furthermore, this mutant mouse model can be used to study the underlying mechanisms of the observed phenotypes.

### 7.2 Regulating Oncogenic Pathway in Glioma

It has been well established that, in cancer cells, alternative splicing patterns change extensively. A comprehensive analysis examining 32 cancer types from 8705 patients demonstrated widespread alternative splicing changes in cancer cells (Kahles et al. 2018). Focusing on high‐grade gliomas (HGG), a more recent study revealed several altered alternative splicing events in HGG cancer‐driver genes (Siddaway et al. 2022). Of these events, *NF1* exon 23a ranked the highest in the significant alternative exons list, showing the biggest change of splicing in HGG compared to normal brain cells. Importantly, increased inclusion of NF1 exon 23a correlated with increased RAS/MAPK activity in HGG and worse patient survival, highlighting the significance of the changed splicing pattern of this exon (Siddaway et al. 2022).

In this study, the RNA‐seq data from 64 HGG patients and 20 normal brains were analyzed. It was found that the inclusion of *NF1* exon 23a was increased in more than 80% of HGG. As discussed in the previous section, exon 23a is normally predominantly skipped in the brain. However, in HGG, the exon inclusion elevated from 20% to 25% to 70%–80%, with a mean increase of 46%, which is a very significant change. It should be noted that in HGG, the *NF1* expression levels remained similar to that in the normal cells, indicating that the splicing pattern change was independent of transcription of the *NF1* gene. When the same analysis was conducted using the TCGA diffuse glioma dataset that included more than 700 glioma samples, a mean increase of 50%–62% was observed for exon 23a expression. Finally, similar results were seen in three HGG mouse models that carry mutations in histone H3 genes (Siddaway et al. 2022).

To further determine the functional significance of the increased NF1 exon 23a inclusion in HGG, morpholino oligos were used to block both ends of exon 23a in pediatric and adult HGG cells to block inclusion of this exon. A striking result was observed: phosphorylated ERK1/2, which is downstream of RAS, was reduced more than 50% in morpholino treated cells than in untreated cells (Siddaway et al. 2022).

Having established that the splicing pattern change of *NF1* exon 23a functioned as a HGG cancer splicing driver, Siddaway and colleagues next investigated the upstream events that led to the splicing change. They tested the expression level of all the splicing regulators that were shown to regulate inclusion of exon 23a (Figure 4) in HGG. They found a strong correlation of decreased expression of the negative regulator family proteins CELF 3–6 and ELAVL2‐4 and a moderate correlation of increased expression of the positive regulators TIA1, indicating that changes in CELF 3–6 and ELAVL2‐4 are the main regulators responsible for the splicing pattern change of exon 23a (Siddaway et al. 2022). These results indicate that decreased expression of CELF and ELAVL2‐4 tips the balance of exon 23a inclusion scale to the right side (Figure 4B) in favor of exon 23 inclusion.

As only 14% of the HGGs in this study have mutations in CELF and ELAVL genes, there had to be non‐mutagenic mechanisms that could explain the decreased expression of CELF and ELAVL in HGG. A search for the transcription factors that bind at the promoter region of the CELF and ELAVL genes led to the identification of RE1 silencing transcription factor (REST). REST was significantly upregulated in HGG, which correlated with repression of CELF and ELAVL genes. Furthermore, siRNA knockdown of REST in primary HGG cells increased the expression of CELF3, CELF4, and ELAVL3 and a more than 50% decrease in *NF1* exon 23a inclusion (Siddaway et al. 2022).

**FIGURE 5:**
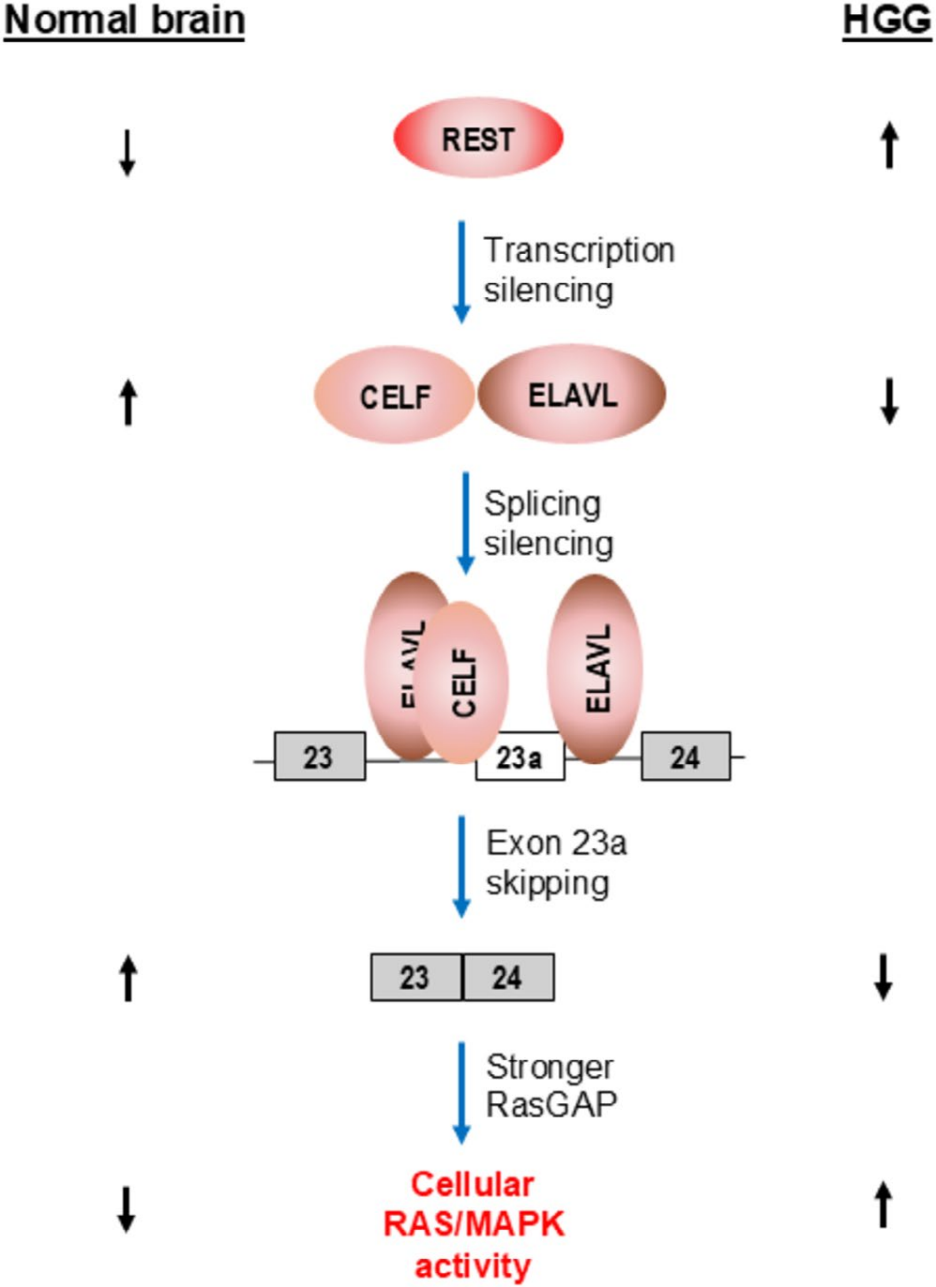
An alternative splicing‐driven oncogenic pathway in HGG. The diagram depicts how alternative splicing of *NF1* exon 23a is regulated in HGG (right), as opposed to what happens to the normal brain (left), that contribute to the increased RAS/MAPK activity. In HGG, expression of the transcription silencer REST is increased, which leads to down‐regulations of CELF and ELAVL proteins, two groups of negative regulators of exon 23a inclusion. As a result, exon 23a is switched from predominate inclusion to skipping, which activates the RAS/MAPK pathway.

This comprehensive study established an oncogenic pathway in HGG that inactivates the NF1 function through altered alternative splicing of exon 23a, which leads to an increased oncogenic Ras/MAPK process (Figure 5).

## 8 Summary and Future Directions

Exon 23a turns out to be a key regulatory exon in NF1 that strongly modifies the RasGAP function of neurofibromin. Inclusion or skipping of this 63‐nt exon changes the functional output of neurofibromin without changing transcription of the *NF1* gene. The biological process controlled by this alternative splicing event has been demonstrated in brain functions and tumor progression. Given the critical role of RAS activity in many aspects of biology, we anticipate many additional biological processes to be discovered that are controlled by this splicing event.

Because changing the splicing pattern of *NF1* exon 23a can offer a quick response to the cellular needs, it is reasonable to postulate that alternative splicing of exon 23a undergoes dynamic regulation responding to environmental stimuli and stress. For example, our study using cardiomyocytes demonstrated that calcium levels change the alternative splicing pattern of exon 23a robustly (Sharma, 2017). It is highly possible that many other environmental stimuli can change the splicing pattern of this mighty, small exon.

## Author Contributions

**Hua Lou:** writing – original draft (equal), writing – review and editing (equal). **Annabelle G. Elsner Pacheco:** writing – original draft (equal), writing – review and editing (equal).

## Conflicts of Interest

The authors declare no conflicts of interest.

## Related WIREs Articles

Splicing alterations in healthy aging and disease

## Data Availability

Data sharing is not applicable to this article as no new data were created or analyzed in this study.
